# Hyperspectral terahertz imaging with electro-optic dual combs and a FET-based detector

**DOI:** 10.1038/s41598-020-71258-6

**Published:** 2020-09-02

**Authors:** Pedro Martín-Mateos, Dovilė Čibiraitė-Lukenskienė, Roberto Barreiro, Cristina de Dios, Alvydas Lisauskas, Viktor Krozer, Pablo Acedo

**Affiliations:** 1 Electronic Technology Department, Universidad Carlos III de Madrid, Leganés, Spain; 2 Physikalisches Institut, Goethe Universität Frankfurt, Frankfurt, Germany; 3 Institute of Applied Electrodynamics and Telecommunications, Vilnius University, Vilnius, Lithuania; 4 Institute of High Pressure Physics PAS, CENTERA Laboratories, Warsaw, Poland; 5 Ferdinand-Braun-Institut, Leibniz Institut für Höchstfrequenztechnik, Berlin, Germany

**Keywords:** Characterization and analytical techniques, Imaging and sensing

## Abstract

In this paper, a terahertz hyperspectral imaging architecture based on an electro-optic terahertz dual-comb source is presented and demonstrated. In contrast to single frequency sources, this multi-heterodyne system allows for the characterization of the whole spectral response of the sample in parallel for all the frequency points along the spectral range of the system. This hence provides rapid, highly consistent results and minimizes measurement artifacts. The terahertz illumination signal can be tailored (in spectral coverage and resolution) with high flexibility to meet the requirements of any particular application or experimental scenario while maximizing the signal-to-noise ratio of the measurement. Besides this, the system provides absolute frequency accuracy and a very high coherence that allows for direct signal detection without inter-comb synchronization mechanisms, adaptive acquisition, or post-processing. Using a field-effect transistor-based terahertz resonant 300 GHz detector and the raster-scanning method we demonstrate the two-dimensional hyperspectral imaging of samples of different kinds to illustrate the remarkable capabilities of this innovative architecture. A proof-of-concept demonstration has been performed in which tree leaves and a complex plastic fragment have been analyzed in the 300 GHz range with a frequency resolution of 10 GHz.

## Introduction

Terahertz (THz) spectroscopy and hyperspectral imaging systems are expected to have an important impact in the sorting industries (food, waste management, etc.)^1^, security^2^ and non-destructive testing^3^. THz spectrometers have demonstrated remarkable capabilities in applications spanning from the detection of foreign bodies, adulteration or microorganisms in food^4^, to explosive compound identification^2^. Furthermore, THz waves easily penetrate packaging materials such as cardboard or plastic enabling the determination of the spectral characteristics of the items inside. Nonetheless, this ever-growing field still holds much promise for an increasing number of applications, including basic research and biomedicine^5^. Indeed, many other uses will surely emerge as higher performing THz systems become readily available. This will involve, as could not be otherwise, the development of superior THz sources and detectors.

The most widely available THz spectral characterization systems nowadays are based on optoelectronic THz generation. With regard to the fundamentals of operation, these systems can be divided in two main architectures: time-domain and frequency-domain^6,7^. Probing a THz spectrum in the time domain, time-domain spectroscopy (TDS), employs pulsed laser sources allowing for more than 6 THz of bandwidth^8^. However, these THz TDS systems traditionally require excessive time for data acquisition due to the time delay method usually employed (even though this problem is being overcome by the use of femtosecond lasers^9^). Besides this, a limited spectral resolution^10^ in comparison to other approaches is provided. On the other hand, the frequency-domain approach is based on photonic continuous-wave (CW) difference frequency THz generation and employs photoconductors covering the frequency range of up to almost 3 THz^11^. This method provides a far higher spectral resolution and accuracy, and a roughly similar dynamic range. In terms of spectral acquisition speed, frequency-domain systems are limited by the thermal wavelength tuning of the lasers.

Besides the long-established optoelectronic THz generation methods mentioned above, nowadays markedly different novel technologies are being developed. For example, solid-state electronic terahertz sources based on direct signal generation oscillators^12^ and also on synthesized signal generators with frequency multipliers using either diode technology^13^ or MMIC technology^14^. These sources exhibit very high spectral resolution, very fast tuning capabilities and output power levels exceeding those of photonic systems. They are restricted nevertheless to a limited frequency bandwidth due to the different waveguide bands. THz systems of up to 2.7 THz have been demonstrated with fully electronic signal sources^15^.

THz signal generation in the frequency range beyond 2 THz can be obtained with quantum-cascade laser (QCL) sources, typically with a limited tuning range and providing maximum power levels in the hundreds of milliwatts^16^. Although many challenges still have to be faced to reach the same level of maturity as solid state THz generators, THz QCLs are a promising approach especially at higher THz frequencies with a performance that is expected to noticeably improve in the decades to come.

Common to the above mentioned novel approaches is the generation of a single narrow signal spectral line, which can then be tuned across a certain bandwidth. Nevertheless, for a THz spectroscopic system would be highly advantageous to probe the whole spectrum of frequencies simultaneously. In this regard, dual-comb^17^ THz systems are rapidly maturing and now hold strong promise for greatly enhancing the performance of current THz spectrometers by providing the simultaneous characterization of the whole spectrum, fast spectral acquisition and very high power spectral density. Several THz dual-comb generation technologies are being currently explored including electronic generation^18^, architectures based on terahertz quantum cascade lasers^19,20^, and photonic-based systems based on mode-locked lasers^21^ and electro-optic combs^22^. Previous THz dual-comb hyperspectral imaging demonstrations include references^23–26^. In particular, hyperspectral imaging with electro-optic THz dual-comb sources^27,28^ has not been demonstrated yet. Nevertheless, electro-optic THz sources provide unique benefits that are unattainable by other technologies, such as the ability of generating combs with ultra-stable repetition rates and offset frequencies (that can be easily referenced to an atomic clock). In addition, the overall signal power can be fully exploited in the desired frequency bands by freely adjusting the spectral coverage of the THz source. Therefore, the scientific potential provided by a hyperspectral imaging architecture based on these sources would be considerable.

On the detection side, THz detection by FET detectors (TeraFET) is becoming a rather established technology relying on FET devices in Silicon or III-V compound semiconductors. It has been demonstrated that FET based THz detectors are able to operate continuously from 100 GHz–2.2 THz with state-of-the-art performance^29,30^ and still operate up to 9 THz^31^.

In this contribution, we demonstrate a novel THz hyperspectral imager based on an electro-optic THz dual-comb generator and a THz FET signal detector that uses 90-nm CMOS technology. The image acquisition is accomplished using raster scanning with a tailored optoelectronic dual-comb that enables the frequency-multiplexed spectral characterization of 2D samples with absolute frequency accuracy, and adjustable resolution and span. A resonant FET-based THz detector operating around 300 GHz with an integrated slot antenna with impedance-transforming dipoles was used for the optimal detection^32^ of the THz dual-comb signal.

## Methods

**Figure 1:**
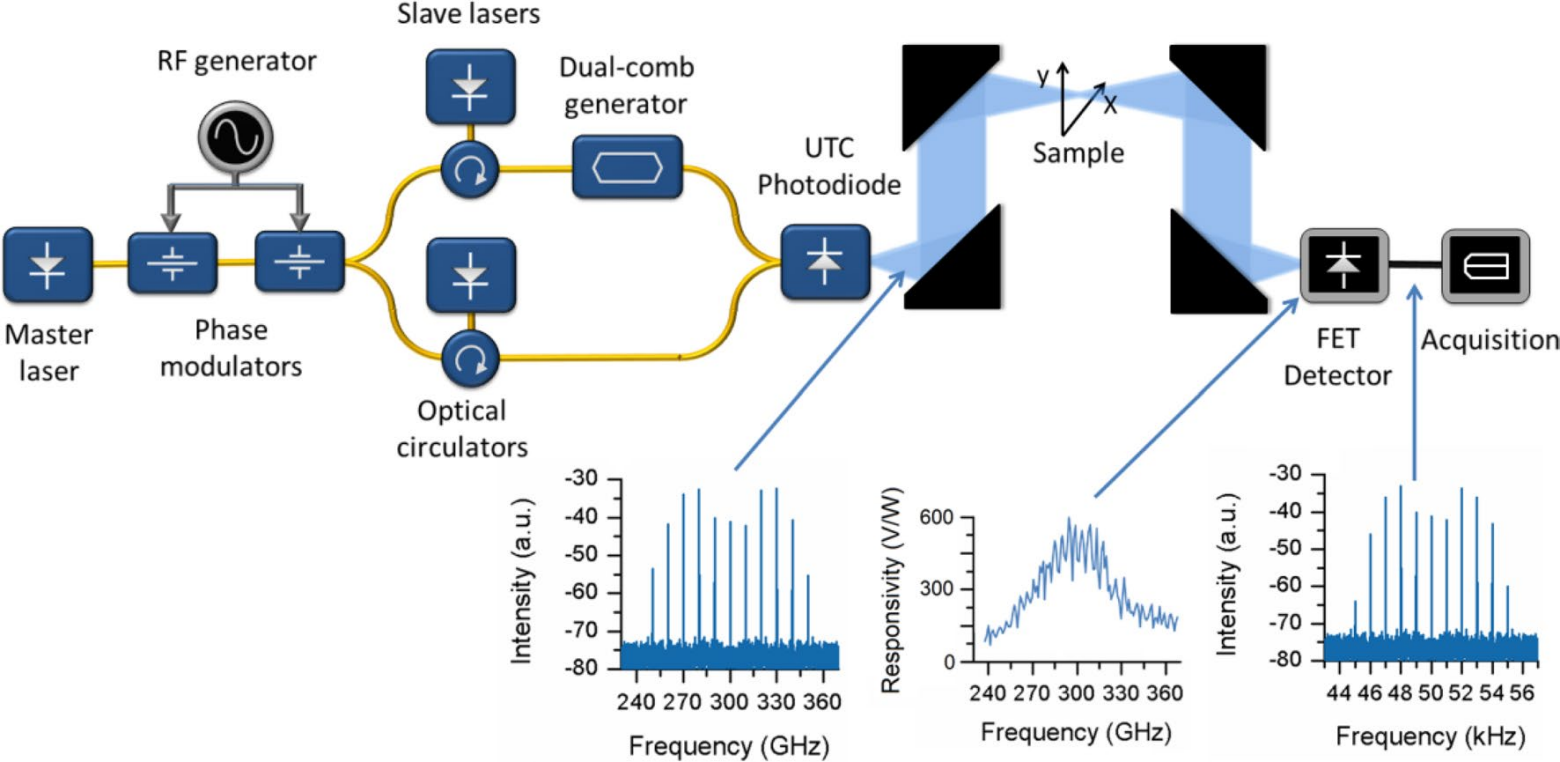
Block diagram of the terahertz dual-comb imaging system. A first optical frequency comb is generated from the output of the master laser by two phase modulators, then two teeth (with a frequency spacing equal to the central THz signal to be generated) are filtered by optical injection locking and a dual-comb signal is created from one of them; both signals are recombined on an UTC photodiode. The emitted THz signal is focused on the sample plane and detected later by a FET detector. The insets show (from left to right) the spectrum of the THz dual-comb, the responsivity of the FET detector and the spectrum of the multi-heterodyne signal after detection.

The simplified architecture of the hyperspectral THz imager is presented in Fig. 1. The terahertz dual-comb generation system is a major evolution of the set up presented in^22^; featuring now improved sensitivity and stability, and superior frequency coverage and resolution. This has been made possible by the complete rearrangement of the fiber optic routing, the selection of higher performing RF components and lasers and the use of an uni-travelling carrier photodiode. Besides this, this paper also proves the experimental applicability of field-effect transistor-based THz detectors for multiheterodyne spectroscopy and imaging.

The architecture of the THz dual-comb source is based on a master optical frequency comb generated from the strong non-linear modulation of a monochromatic continuous wave optical signal (master laser). Therefore, phase coherent optical comb teeth with a separation equal to the RF modulation frequency are generated. The phase of the RF signal applied to each modulator has to be adjusted for maximum spectral span and flatness. A well-known method for the generation of THz waves is based on filtering two comb lines, which are separated by the exact desired THz frequency and beating them on an ultra-fast photodetector^33,34^. In this way, a high-quality and frequency accurate terahertz signal is generated. In the set up illustrated in Fig. 1, this very same procedure is employed: two optical circulators inject the master comb into the cavity of two slave lasers. Hence, two teeth of the master comb are isolated (freely selectable within the wavelength range of the slave lasers) by optical injection locking. In contrast with this traditional CW THz generation method, the architecture shown in Fig. 1 generates a THz dual-comb by creating an electro-optic dual-comb^27,28,35,36^ from one of the two filtered teeth. The resulting signals are heterodyned on a fast photodiode to generate the final THz dual-comb^22^ which is sent through the sample to perform spectral interrogation. The interference signal read by the FET THz detector is digitized and processed to extract the spectral profile of the sample at each spatial location. Overall, this approach provides flexibility in the configuration of the central frequency, the distance between teeth and the overall number of teeth of the THz dual-comb, allowing for the optimization of the available signal-to-noise ratio (refer to Ref.^22^ for details). Besides this, the THz signals inherit the high mutual coherence between combs that characterize electro-optic dual-comb sources, enabling straightforward signal detection and data processing without complicated inter-comb synchronization mechanisms.

For the implementation of the THz dual-comb source, a 1,550 nm narrow linewidth diode laser (EP1550-0-NLW, Eblana Photonics) and two 10 GHz lithium niobate phase modulators (PM-5SES-10-PFA-PFA-UV, EOSPACE) in series have been employed to generate the master comb. The laser was driven by a low noise current source (LDC/E-Currentx00, Luz WaveLabs) and a high resolution temperature controller (LDC/E-Temp3, Luz WaveLabs). A low-phase noise signal generator (MG3692C, Anritsu), a 3 dB RF splitter, a RF phase shifter and two RF power amplifiers were employed to drive the optical modulators. The master optical frequency comb is split and taken to two optical isolators and two variable optical attenuators, which allow for power injection ratio optimization. The slave lasers, with central wavelengths of 1548.8 and 1551.2 (Eblana Photonics), were also driven by low noise controllers. At the output of the circulators the two coherent lines of the master comb are already filtered. In the experiment presented below, a 15 GHz modulation signal was applied to the master comb modulators and the slave lasers were configured to filter the 20th harmonic in order to generate a frequency of 300 GHz, which has been set as the central frequency of the THz dual-comb. As presented above, the THz dual-comb system presented here has the highly desirable characteristic of being adaptable to the frequency range of interest. Subsequently, the output of the circulator connected to the 1551.2 nm laser is injected into the electro-optic dual-comb generator. This system follows a traditional design comprised of a 3 dB optical splitter, two 40 MHz frequency shifters (acousto-optic modulators) (T-M080-0.5C8J-3-F2S, Gooch and Housego), two additional phase modulators with external RF termination (PM-5SES-10-PFA-PFA-UV, EOSPACE) and a final optical coupler. While the acousto-optic modulators are driven at an offset frequency of 50 kHz by a RF synthesizer (HS9004B, Holzworth Instrumentation), the two combs exhibit a spectral signal spacing of 10 GHz and a difference in repetition rates of 1 kHz (the modulation signals are generated by a APMS20G-2, AnaPico), which can be depicted in the insets of Fig. 1. The resulting dual-comb is then amplified and combined with the 1548.8 nm line. This signal is finally applied to a uni-travelling carrier photodiode (IOD-PMAN-13001, NTT Electronics) that is placed on a three-axis translation stage for accurate positioning. The multiple beating products in the photodiode produce a terahertz dual-comb signal with a central frequency of 300 GHz and a separation between lines of 10 GHz, 9 spectral lines were generated to match the available bandwidth on the detector. As shown in Fig. 1, once in free space, the THz signal is first focused into the image plane by a 100 mm and a 150 mm parabolic mirrors and then into the detector using two additional 150 mm parabolic mirrors. The measured beam waist (spatial resolution) has been estimated to be 1.5 mm. A two axis linear translation stage (Standa) is employed to scan the sample. The system is completed, on the detection side, with a THz CMOS detector with an integrated slot antenna and impedance-transforming elements (optical sensitivity above 55 kV/W and an optical NEP < 20.8 pW/Hz^1/2^ at a full 3-dB bandwidth of 42 GHz; the responsivity can be found in the inset of Fig. 1)^32^. The detected signal was amplified using a low-noise amplifier with gain in excess of 40 dB and operated from a battery to avoid any additional 1/f noise at the output of the detector. The detector noise is limited only to the thermal noise in the whole 3-dB bandwidth of 200 kHz, except from frequencies below 10 Hz, due to the low 1/f noise of the amplifier. The output from the amplifier is then connected to a multichannel lock-in amplifier implemented in software taking advantage of a PXI acquisition platform (PXIe-1082 chassis and PXI-5105 acquisition card, National Instruments) that measures the amplitudes of the individual teeth of the comb. Besides this, the PXI system also controlled the raster scanner. The dynamic range of the dual-comb system, defined as the average ratio (for all the teeth of the dual-comb) between the individual tooth intensity and the noise floor, has resulted in 55 dB for an integration time of 200 ms. The signal-to-noise ratio has also been calculated as the mean teeth intensity divided by the standard deviation in ten consecutive measurements averaged for all the comb limes; yielding a signal-to-noise ratio equal to 89 for an integration time of 200 ms when a sample with a transmittance of 0.15 is analyzed.

## Results

For the measurements that follow, a square area of 32 mm was scanned in 1 mm steps to obtain a hypercube with 32 by 32 positions and 9 spectral bands (from 260 to 340 GHz in 10 GHz steps). Each spectral characterization is the result of an average of 10 consecutive measurements with an integration time of 200 ms each. The acquisition time of the whole hypercube is of approximately one hour. Several proof of principle experiments have been carried out to validate the performance of the proposed hyperspectral imaging system.

**Figure 2:**
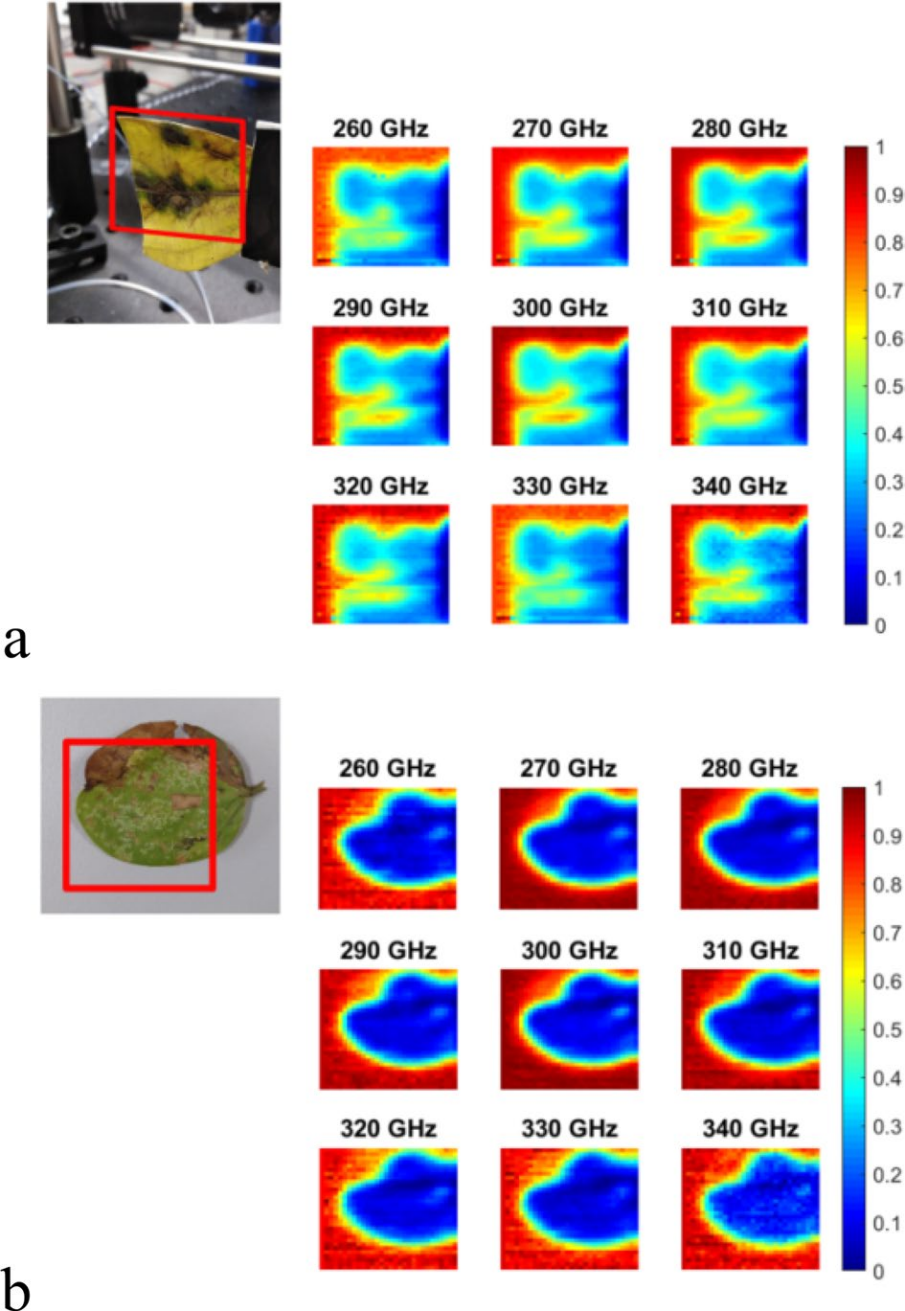
Photographs of different tree leaves (Fabaceae family) together with the transmittance characterized at frequencies going from 260 to 340 GHz. The red squares highlight the analyzed area. All the images are normalized to a maximum value of 1.

**Figure 3:**
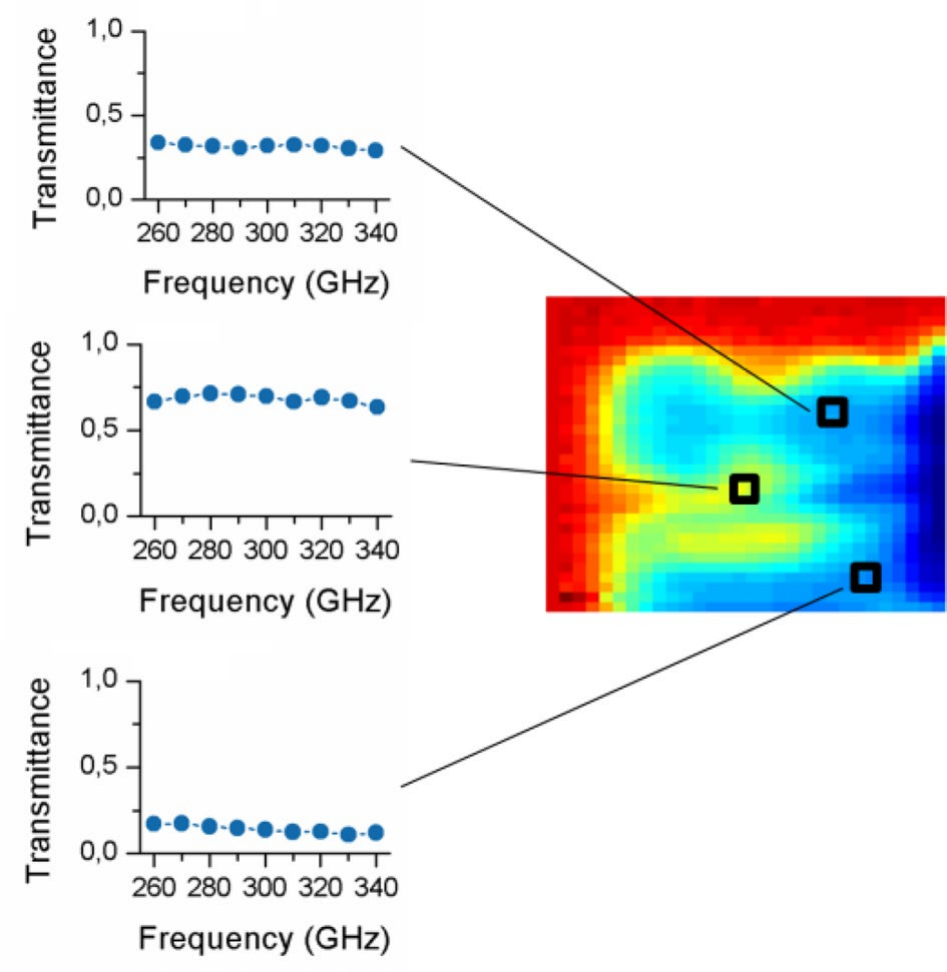
THz spectra at several locations of the sample (tree leaf). The insets show the spectral response at various spatial positions; spectral differences are evident.

Firstly, the spatial and spectral characterization of different tree leaves (Fabaceae family) was performed. Figure 2a shows a photograph of the actual sample (the red square represents the measuring area) together with the acquired hyperspectral images showing the measured transmittance at each frequency component (signal intensity for all the spectral lines is normalized to 1). It can be seen that the various regions in the sample exhibit clear differences in spectral transmittance, which could be due to the biological tissue and/or water content variations. The results of a second test, in which a different leaf of the same family is analyzed, can be found in Fig. 2b. In this case, a clear region around the leaf is visible with increased transmittance as compared to the leaf central part, which would in principle indicate either reduced water absorption or, more probably, edge diffraction effects. Both characterizations demonstrate the capabilities of the proposed architecture for providing spectrally resolved images. To further illustrate this point, Fig. 3 shows the spectral analysis at three particular locations of the sample in Fig. 2a; clearly distinctive spectral responses can be discerned. While the top and bottom locations exhibit a similar spectral trend, the location in the center of the leaf shows a certainly dissimilar response. This indicates, very likely, a difference in biological tissue structure that goes beyond the hydration level. Indeed, the spectral information offered by the system should enable in the very near future to isolate the impact of the hydration state from other effects such as permittivity changes in the sample due to other physiological variations.

**Figure 4:**
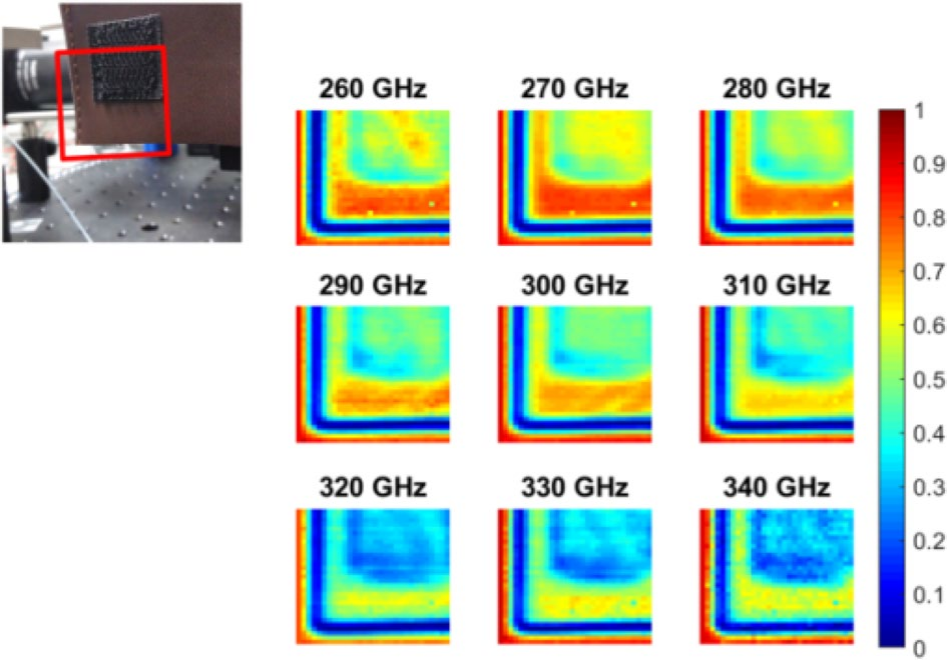
Results of the characterization of a complex plastic sample. The normalized transmittance is represented at the 9 interrogation frequencies of the dual-comb source.

**Figure 5:**
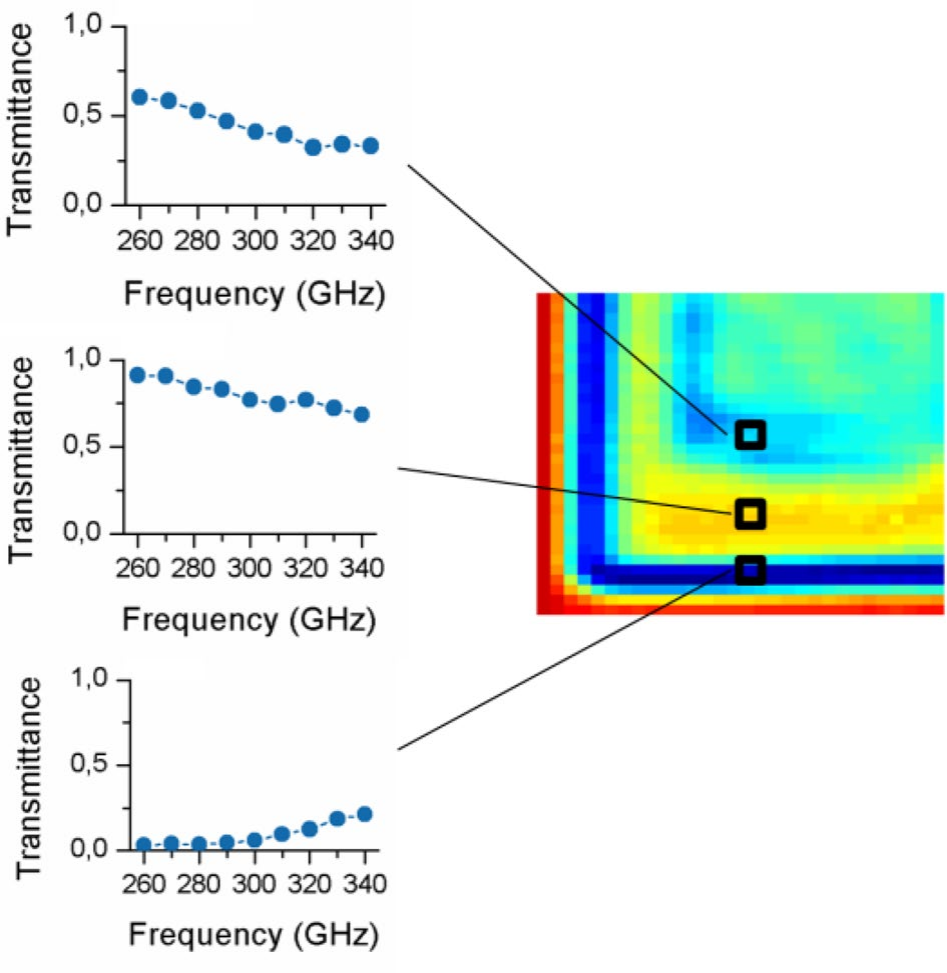
The transmittance spectra at several locations enables a straightforward separation between plastic layers (differences in the overall absorbance and the frequency dependent slope) and the edge diffraction.

The hyperspectral THz imager has also been tested as an inspection tool for industrial manufacturing. To that aim, a fragment of a soft plastic case, shown in Fig. 4, has also been characterized. The hyperspectral images exhibit an utterly clear differentiation between the different structures within the sample. Although in general there is a characteristic quasi-linear trend in which losses increase for higher frequencies, the transmittance of the upper right half of the sample is much higher, also presenting a more pronounced slope. Interestingly, diffraction at the edge of the sample produces a significant reduction in the signal that reaches the terahertz detector. These effects are apparent in Fig. 5, where we can spectrally differentiate between different components of the sample and edge diffraction, (which is not only determined by the material properties but also by the geometrical characteristics). The capabilities of this approach are obviously far higher than those of single frequency arrangements, and could be of extraordinary importance for nondestructive testing and quality control monitoring scenarios.

## Discussion and conclusion

The THz hyperspectral imaging system presented in this paper has demonstrated its feasibility and effectiveness for the hyperspectral analysis of various biological and artificial samples. The main advantage of the THz dual-comb approach over frequency sweep based sensing systems is that the spectral information is retrieved simultaneously and coherently for all the frequency points, which results in a parallel detection of the spectral properties, as well as a faster, more consistent measurement. Additionally, the THz illumination signal can be tailored with flexibility (center frequency, frequency resolution and frequency span) to the requirements of a particular experiment or receiver to maximize the spectral resolution and the signal-to-noise ratio.

The architecture presented here can be configured to generate and detect THz combs in a frequency range that goes from a few tens of GHz up to roughly 1.2 THz with freely adjustable optical resolution (down to a single Hz^22^) and span, exceptional simplicity and an inherently high mutual coherence that enables integration times of up to few hundreds of a second without active stabilization. The main limitation of the current system is evidently the long time needed for the acquisition of the hypercube; nevertheless, recent dual-comb developments promise to overcome current technological challenges to consistently shrink acquisition times to a single second in the next few years^37,38^. Besides this, we plan to incorporate spectral processing and classification to the next versions of the system for the identification and quantification of analytes with distinctive absorption spectrum^39^.
